# Corticobasal Syndrome Associated with Antiphospholipid Syndrome Secondary to Systemic Lupus Erythematosus

**DOI:** 10.1155/2018/5872638

**Published:** 2018-05-07

**Authors:** Ritsuo Hashimoto, Tomoko Ogawa, Asako Tagawa, Hiroyuki Kato

**Affiliations:** Department of Neurology, International University of Health and Welfare Hospital, Tochigi, Japan

## Abstract

We report the case of a 53-year-old woman diagnosed with corticobasal syndrome (CBS) due to antiphospholipid syndrome (APS) secondary to systemic lupus erythematosus. Brain MRI showed marked cortical atrophy, several small infarctions in the deep white matter, and mild white matter changes, all of which were probably due to thrombosis manifestations of APS and could also be related to the CBS. To the best of our knowledge, this is the fourth reported case of CBS due to APS. It is noteworthy that although the common underlying pathologies of the CBS are neurodegenerative diseases, either primary or secondary APS can manifest itself as the CBS.

## 1. Introduction

Corticobasal syndrome (CBS) is characterized by asymmetric involuntary movements including rigidity, dystonia, tremor, and myoclonus combined with cortical symptoms such as apraxia, cortical sensory loss, and alien limb phenomena [1]. Its underlying pathologies reported so far are diverse and heterogeneous; they include corticobasal degeneration, progressive supranuclear palsy, Alzheimer's disease, Pick's disease, Lew body dementia, motor neuron inclusion body dementia, dementia lacking distinctive histopathology, and Creutzfeldt-Jakob disease [2–4]. In this report, we present a rare case in which a patient developed CBS due to antiphospholipid syndrome (APS) [5–7].

## 2. Case Report

A 53-year-old right handed woman was referred to our hospital to treat progressive parkinsonism. The patient had experienced convulsions 3 years before her first visit to our hospital and was on medication (diphenylhydantoin 200 mg/day). She developed akinetic-rigid syndrome (dominant on the left side) and a shuffling gait several months after experiencing convulsions. The previous doctor prescribed L-dopa, which proved to have little effect on her condition. The patient had also demonstrated livedo reticularis of both forearms around the time she presented the akinetic-rigid syndrome. There was no personal history of venous or arterial thrombosis or miscarriage.

On admission, the patient was alert and cooperative, yet she appeared to be slightly depressed. Her speech was easily understandable, albeit slightly hypophonic. Upon examination, she had mild supranuclear gaze palsy showing slight difficulty in looking down. Her horizontal pursuit eye movements were slightly saccadic while the range of motion was normal. Motor power was intact, while exaggerated tendon reflexes were noted on the left side with positive jaw reflex. Babinski sign was negative on either side. Snout reflex was present, yet grasp and palmomental reflexes were negative. There was asymmetric upper limb akinesia and rigidity which was more severe on the left side than on the right. Also, dystonia of the left arm and leg was observed. The gait was slow and stiff with absent arm swing, yet her postural reflexes were relatively well preserved. The patient showed bilateral limb-kinetic apraxia which was prominent on the left side. She could barely use fingers in her right hand to show patterns such as victory sign; however, it was impossible to do so with her left hand. Miming the use of tools and habitual movements such as using a toothbrush, beckoning, and indication of getting away were also difficult for her especially with the left hand which was probably due to the limb-kinetic apraxia. Primary sensations were intact except for loss of joint position sense at her left elbow. There were no cortical sensory deficits such as agraphesthesia and astereognosis. The alien limb phenomenon, neglect syndrome, or myoclonus was not observed. Her mini mental status examination (MMSE) score was 20/30 demonstrating disorientation in time, dyscalculia, mild anterograde amnesia, and decreased digit span.

**Figure 1:**
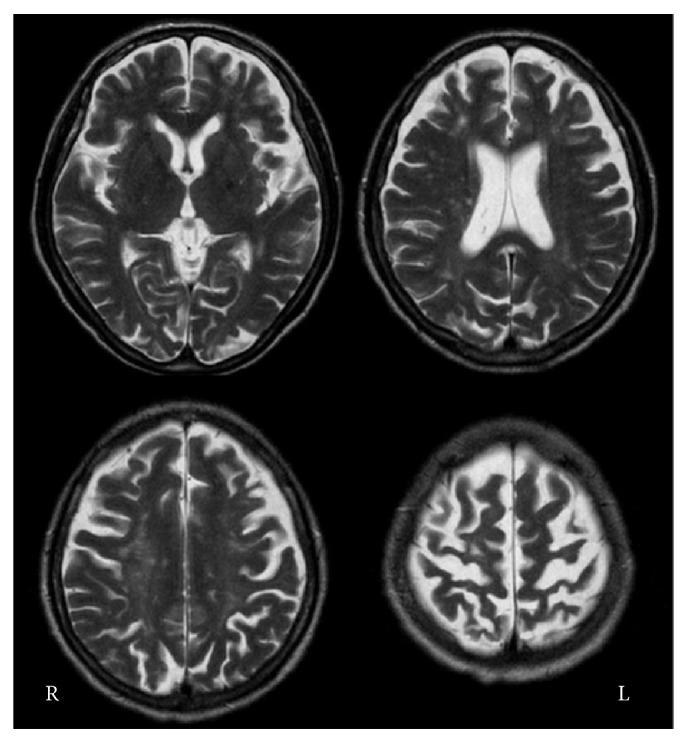
Axial T2-weighted magnetic resonance imaging 2 days after admission showing small infarctions in the deep white matter and subcortical high-signal intensities in both hemispheres. Diffuse cortical atrophy that is more prominent in the central areas is also demonstrated. Note that neither ischemic nor atrophic changes are present in the basal ganglia.

**Figure 2:**
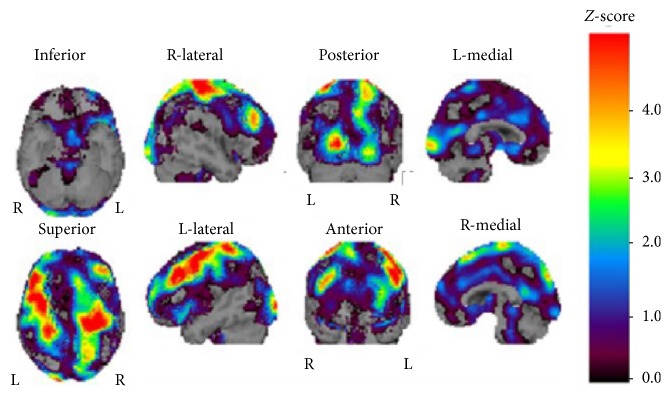
^99m^Tc-ECD SPECT with easy *Z*-score Imaging System 10 days after admission showing decreased cerebral blood flow in the bilateral central areas that extends to the frontal and parietal areas.

Laboratory investigations revealed there were thrombocytopenia, slight increased serum creatinine with no proteinuria, elevated activated partial thromboplastin time (APTT), and positive anticardiolipin antibody with IgG titer 35 (positive > 9). Antinuclear antibody was present at a titer of 1 : 1280 (positive > 79), and anti-DNA antibody was also positive at a titer of 11.7 (positive > 6.0). Test result for SS-A and SS-B antibodies was negative. Complement levels were normal (Table 1). The serological abnormalities including positive anticardiolipin, antinuclear, and anti-DNA antibodies remained, as observed in subsequent tests performed after 3 months. Anticardiolipin antibody was detected in two different times; APS diagnosis was therefore performed. In addition, the present case fulfilled the American College of Rheumatology (ACR) criteria for diagnosing systemic lupus erythematosus (SLE) [8]; she manifested neurological symptoms (convulsions and dementia), and her laboratory data showed hematological involvement (thrombocytopenia), immunological disorder (positive anticardiolipin antibody) and positive antinuclear and anti-DNA antibodies. Brain MRI showed subcortical white matter ischemic changes, marked cortical atrophy that was prominent in the bilateral central areas, and several small infarcts in deep white matter of both hemispheres (Figure 1). Single photon emission tomography using technetium-99m-L, L-ethyl cysteinate dimer (^99m^Tc-ECD SPECT) showed decreased cerebral blood flow in the bilateral central areas, which extended to the frontal and parietal areas (Figure 2).

A diagnosis of CBS due to APS secondary to SLE was made, and aspirin treatment was initiated. Because the patient showed no active systemic disorder other than the neurological manifestations, no therapy was introduced due to SLE. During follow-up, diphenylhydantoin was replaced by zonisamide; however, anticardiolipin, antinuclear, and anti-DNA antibodies tested positive consistently suggesting that the SLE was not induced by diphenylhydantoin. Dabigatran etexilate (75 mg/day) was administered in addition to aspirin at a time; however, APTT prolonged over 3 times that of the contorls' and was discontinued. The patient presented left side dominant akinetic-rigid syndrome and limb-kinetic apraxia over the 8-year follow-up period, both of which changed little in terms of severity. She was ambulant until 61 years of age when she suffered from compression fracture of the 9th thoracic vertebra, which bound her to wheelchair. Repeated MRI scans performed during the follow-up period demonstrated slight progression of diffuse cortical atrophy and subcortical ischemic changes; however, no new infarction was noted. At the age of 62, she died from pneumonia at a local hospital. A postmortem study was not obtained.

## 3. Discussion

A diagnosis of CBS was made in the present case based on clinical manifestations including left side dominant akinetic-rigid syndrome and limb-kinetic apraxia, both of which were insidious at onset [1]. This case represents the fourth reported description of CBS due to APS. All the previously reported cases had primary APS (APS exists as an independent condition) [5–7]; however, the APS described in the present case was secondary to SLE (Table 2). Thus, this was the first case of CBS due to APS secondary to SLE.

The types of brain MRI lesions observed in the three cases reported previously are as follows: case 1 [5] had multiple infarcts in the cerebral hemispheres and basal ganglia with prominent lesions in the right parietal lobe and head of the left caudate nucleus; case 2 [6] showed extensive white matter changes, marked diffuse cerebral corticosubcortical atrophy, and several infarcts in both hemispheres, involving multiple vascular territories, including the striatum bilaterally; and case 3 [7] demonstrated only diffuse brain atrophy without evidence of cerebral infarction; dopamine transporter imaging studies were normal. Our case had marked cortical atrophy, several small infarctions in the deep white matter, and mild white matter changes; however, neither infarction nor atrophy was noted on either side of the striatum (Table 2). Our case implies that the akinetic-rigid syndrome associated with APS may not necessarily indicate ischemic pathology of the basal ganglia and it may be due to cortical pathology. Overall, it is suggested that in most of the cases APS is related to thrombosis manifestations and could also be related to the CBS. An exception is case 3 [7] who demonstrated only diffuse brain atrophy without evidence of cerebral infarction. The presence of an undetermined antibasal ganglia antibody cannot be ruled out [7, 9].

With regard to the treatment, all patients received anticoagulation and/or antiplatelet treatment. In case reported previously, stabilization of the symptoms was observed in two patients (cases 1, 3) following treatment [5, 7], but one patient (case 2) showed no significant improvement and developed a new infarct at the follow-up performed after 2 years [6]. The patient described in this report exhibited no obvious progression in the symptoms of CBS when using aspirin during the 8-year follow-up. These observations imply that anticoagulation and/or antiplatelet treatment might be effective in preventing progression of symptoms at least in some patients, even if not efficient enough to alleviate them.

In summary, we have reported the fourth case of CBS associated with APS. It must be noted that although the common underlying pathologies of the CBS are neurodegenerative diseases, either primary or secondary APS can manifest itself as the CBS.

## Figures and Tables

**Table 1:** Laboratory data of the patient.

WBC	7530/*μ*L	(3500–9700)	Glucose	105 mg/dL	(70–109)
RBC	427 × 10^4^/*μ*L	(376–516 × 10^4^)	HbA1c (JDS)	5.5%	(4.3–5.8)
Plt	11.5 × 10^4^/*μ*L	(14.0–37.9 × 10^4^)	CRP	0.25	(0.00–0.30)
PT-INR	1.05	(0.84–1.14)	CH_50_	44	(30–45)
APTT	46.1 sec	Control: 31.6 sec	C3	113	(80–140)
TP	7.7	(6.7–8.3)	C4	25.9	(11–34)
Alb	4.3	(3.9–4.9)	ANA	1280	(<79)
T-Bil	0.3	(0.2–1.2)	DNA/RIA	11.7 IU/mL	(0.0–6.0)
AST	18 IU/L	(8–38)	SS-A	-	
ALT	7 IU/L	(4–44)	SS-B	-	
BUN	20.2 mg/dL	(8.0–20.0)	aCL-IgG	35 U/mL	(0–9)
Cr	0.85 mg/dL	(0.47–0.79)	Urinalysis		
T-CHO	240 mg/dL	(150–219)	Protein	-	
HDL-C	62 mg/dL	(40–90)	OB	-	
Na	142 mEq/L	(135–145)			
K	4.3 mEq/L	(3.5–5.0)			
Cl	105 mEq/L	(98–108)			

( ), normal range; ANA, antinuclear antibody; DNA/RIA, anti-DNA antibody/radioimmunoassay; aCL-IgG, anticardiolipin antibody-IgG; OB, occult blood.

**Table 2:** Reported cases of corticobasal syndrome associated with antiphospholipid syndrome.

Case	Authors	APS	Age/sex	Imaging studies	Treatment	Follow-up (period)
1	Lees and Morris [5]	primary	44/F	MRI: multiple infarcts in the cerebral hemispheres and basal ganglia with prominent lesions in the right parietal lobe and head of the left caudate nucleus	Warfarin and aspirin	Moderate improvement in apraxia (3 years)

2	Martino et al. [6]	primary	56/F	MRI: extensive white matter changes, marked diffuse cerebral corticosubcortical atrophy, and several infarcts in both hemispheres involving multiple vascular territories, including the striatum bilaterally	Aspirin and warfarin were separately tried	Progressive deterioration (2 years)

3	Lee et al. [7]	primary	47/M	MRI: only diffuse brain atrophy without evidence of cerebral infarction, and dopamine transporter imaging studies using [^18^F] FP-CIT PET: normal	Warfarin	No change (6 months)

4	Our case	secondary	53/F	MRI: marked cortical atrophy, several small infarctions in the deep white matter, and mild white matter changes, no infarction nor atrophy in the striatum ^99m^Tc-ECD SPECT: decreased cerebral blood flow in the bilateral central area extending into the frontal and parietal areas	Aspirin	No significant change (8 years)

APS, antiphospholipid syndrome; [^18^F] FP-CIT PET, positron emission tomography using ^18^F-fluorinated *N*-3-fluoropropyl-2-*β*-carboxymetholxy-3-*β*-(4-iodophenyl) nortropane; ^99m^Tc-ECD SPECT, single photon emission tomography using technetium-99m-L, L-ethyl cysteinate dimer.
